# Electrospun Membrane for the Extraction of Acrylamide in Pet Food Samples

**DOI:** 10.1155/2021/1285501

**Published:** 2021-10-22

**Authors:** Chanbasha Basheer

**Affiliations:** ^1^Department of Chemistry, King Fahd University of Petroleum and Minerals, Dhahran 31261, Saudi Arabia; ^2^Interdisciplinary Research Centre for Membrane and Water Security, King Fahd University of Petroleum and Minerals, Dhahran 31261, Saudi Arabia

## Abstract

A simple microextraction procedure was developed using an electrospun nanostructured membrane to determine acrylamide in pet food samples. Polyvinyl chloride, polyvinyl alcohol, and polyvinyl alcohol/hydroxyethyl cellulose electrospun membranes were prepared and investigated as a sorbent to extract acrylamide. The characterization of the synthesized electrospun membrane was accomplished using field-emission scanning electron microscopy (FESEM). FESEM images showed uniform morphology and beadless nanofibers. Quantification was done by high-performance liquid chromatography with ultraviolet detection. A series of microextraction parameters were optimized before quantitative analysis of dry pet food samples. The calibration curve exhibited good linearity with a correlation coefficient of 0.996 across a 1–100 *μ*g/kg concentration range. The recovery of acrylamide for pet food samples spiked with 5 and 10 *μ*g/kg was in the range of 79.6–113.9 (*n* = 3). The intraday precision of the method was less than 12% for three replicated real spiked samples at the 5 *μ*g/kg level. The results demonstrated that the electrospun nanostructured membrane has good extraction selectivity and minimal matrix effect with an enrichment factor of 180-fold.

## 1. Introduction

The Swedish National Food administration listed acrylamide as a probable human carcinogen [1]. A large amount of acrylamide is detected in common food products such as French fries, potato crisp, breakfast cereal, and many other baked products. It was first discovered by a research group [2] that the concentration of acrylamide present in a range of fried and oven-cooked foods could go up to 5 mg/kg. It had become one of the most significant controversies in food science ever since. Acrylamide was proven to have mutagenicity in rats, and it has been classified as a probable human carcinogen and a known human neurotoxin [3–5]. The Maillard reaction was believed to be the primary source of acrylamide found in food [6]. It involves the reaction between asparagine and a carbonyl source that will undergo a series of reactions to give acrylamide as a byproduct [7]. Acrylamide is usually found in high-temperature processed carbohydrate-rich and roasted foods [4]. In order to supply an energy source to pets, most dry pet foods are made of high-carbohydrate ingredients such as rice bran, oatmeal, millet, barley, and potatoes, and these foods are often prepared by baking. Previous studies suggested that dry pet foods may likely contain acrylamide [8].

A sample preparation process is necessary before the analysis of dry pet food samples. The concentration of acrylamide in pet food is expected to be low; it is necessary to have a preconcentration step before analysis. A commonly used technique is solid-phase extraction (SPE), which involves a large sample size volume [7]. Various extraction techniques have been reported for the determination of acrylamide in food samples: solid-phase microextraction (SPME) [9] and matrix solid-phase dispersion [10]. The SPME technique has limitations; the fibers are easily fragile and costly [11]. The SPE requires a large amount of sample and solvent, and the cartridges are expensive [8, 12]. Solvent-minimized extraction methods, dispersive liquid-liquid microextraction (DLLME) and liquid-phase microextraction (LPME), are other methods reported for acrylamide in food samples [13, 14]. A major limitation for the solvent-minimized approach is selecting suitable solvents for the polar target analytes [15].

Recently, electrospun polymer nanofiber-assisted extractions are reported in the literature for a wide range of target analytes [16–22]. All these reports showed considerable improvement in the extraction efficiency and recovery with a small sample size.

The main objective of this work is to demonstrate the applicability of the electrospun nanostructure membrane used in the microextraction method. After extraction, the quantification of acrylamide was performed by high-performance liquid chromatography (HPLC).

## 2. Experimental

### 2.1. Chemicals and Reagents

The following polymers were purchased from Aldrich Chemical Company Inc. (Milwaukee, WI, USA): polyvinyl alcohol (PVA) (*M*_*w*_ 85,000–140,000), hydroxyethyl cellulose (HEC) (*M*_*w*_ 250,000), and polyvinyl chloride (*M*_*w*_ 120,000). Acrylamide was purchased from Bio-Rad Laboratories (Richmond, CA, USA). HPLC-grade acetonitrile was purchased from M. Tedia Company Inc. (Fairfield, IL, USA) and sulfuric acid from E. Merck (Germany). Ultrapure water was prepared on a Milli-Q (Milford, MA, USA) system. A standard stock solution of 100 mg/L of acrylamide was prepared in water. A working standard solution at 1 mg/L was used for low-concentration spiking. All solutions were stored at 4°C.

### 2.2. Electrospinning Setup

The electrospinning setup is self-assembled and consists of an electrospun control system, a DW-P403-1AC voltage supply unit, and a TS 2–60 multisyringe pump, all supplied by Optrobio Technologies Pte. Ltd. (Singapore). The collector was a conducting plate spread with aluminum foil for the collection of nanofibers formed.

### 2.3. Fabrication of the Nanostructured Membrane

#### 2.3.1. Polystyrene (*M*_*w*_ 45,000)

Polystyrene solution for electrospinning was prepared by dissolving 50 wt. % PS in tetrahydrofuran (THF), and electrospinning was performed by applying a high voltage of 18 kV between the syringe needle containing the PS solution and the collector. The working distance between the tip of the needle and the collector was 10 cm. The flow rate of the polymer solution was maintained at 50 *μ*L/min.

#### 2.3.2. Polyvinyl Alcohol

Polyvinyl alcohol solution for electrospinning was prepared by dissolving 15 wt. % PVA in water, and electrospinning was performed by applying a high voltage of 20 kV between the needle of the syringe containing the PVA solution and the collector. The working distance between the tip of the needle and the collector was 10 cm. The flow rate of the polymer solution was maintained at 10 *μ*L/min.

In addition to pure PVA nanofibers, it is also interesting to investigate different types of nanofibers formed by combining another compatible polymer with PVA. In this case, hydroxyethyl cellulose (HEC) was chosen; it was prepared by dissolving 5 wt. % HEC in water and mixed with 15 wt. % PVA in a 1 : 1 ratio with continuous stirring for at least 12 hrs, followed by electrospinning using the same condition as above to give PVA/HEC nanofibers.

As PVA is highly soluble in water, an additional step was needed to make the PVA-based nanofibers insoluble in water used for microextraction with water as the medium. The additional step was the cross-linking of PVA nanofibers using glutaraldehyde in the presence of acid for 6 hours. In this additional step, the hydroxyl groups present in PVA and HEC cross-linked to form acetal ring groups or ether linkages, as a result, rendering them insoluble in water.

#### 2.3.3. Polyvinyl Chloride

Polyvinyl chloride solution for electrospinning was prepared by dissolving 20 wt. % PVC in tetrahydrofuran (THF), and electrospinning was performed by applying a high voltage of 20 kV between the needle of the syringe containing the PVC solution and the collector. The working distance between the tip of the needle and the collector was 10 cm. The flow rate of the polymer solution was maintained at 50 *μ*L/min.

### 2.4. Sample Preparation

All dry pet foods were purchased from local pet shops or supermarkets. Five grams of each sample were grounded in a commercial blender and 20 mL of water to obtain a homogenized suspension for 5 min. Another 10 mL of water was added, and the homogenates were left to stand in a water bath under agitation. After 30 min, the homogenates were centrifuged (12,000 rpm, 20 min) to sediment solid particles and filtered through Whatman No. 2 filter paper. The filtrates were transferred to a 50 mL volumetric flask and topped up to the mark with water. Any unused portions were stored at 4°C.

### 2.5. Microextraction Procedure

The initial extraction procedure was carried out on 10 mL of the 50 *μ*g/kg spike sample (acrylamide-free food sample) prepared in a 20 mL sample vial. A piece of nanostructured membrane weighing approximately 5 mg (≈1 × 1 cm) was added to the sample solution together with a magnetic stirring bar. The sample was stirred for 30 min at 300 rpm for extraction to proceed. During extraction, the nanostructured membrane tumbled freely in the sample solution.

After extraction, the nanostructured membrane was taken out from the sample solution with a pair of forceps and placed in a 500 *μ*L microvial together with 100 *μ*L of 0.01 M sulfuric acid desorbed via ultrasonication for 30 min. After desorption was done, the nanostructured membrane was removed. The extract was filtered through a 0.45 *μ*m (13 mm dia.) syringe filter unit before 10 *μ*L was injected into the HPLC system for analysis. All extractions were performed in triplicate (*n* = 3) to calculate the repeatability.

### 2.6. HPLC Analysis

Analysis was carried out on a Shimadzu (Kyoto, Japan) HPLC system comprising an LC6AD binary pump, a DGU-14A degasser, an SPD-20A UV/vis spectrophotometric detector set at 200 nm, and a CBM-20A communication bus module. Data were collected and processed using LC Solution (Shimadzu) data analysis software. A PAH C_18_ column (250 mm × 4.6 mm; 5 *μ*m) from Waters (Milford, MA, USA) was used with a mobile-phase flow rate of 0.6 mL/min. The mobile phase used was a 20–80% acetonitrile-0.01 M sulfuric acid mixture (pH of the mobile phase mixture was adjusted to 2). A column temperature of 25°C was maintained. 10 *μ*L of the sample was injected each time through a manual injector. No derivatization was needed before injection.

## 3. Results and Discussion

### 3.1. Morphology of the Nanostructured Membrane

**Figure 1:**
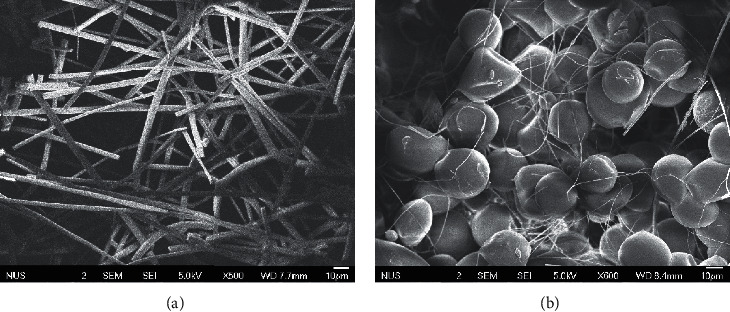
Scanning electron micrographs of PS nanofibers: (a) short and discontinuous and (b) beaded structures.

The nanostructured membranes have a porous network with lightweight, making them suitable as a sorbent material for microextraction. Morphologies were observed under a JEOL JSM-6701F field-emission scanning electron microscope (FESEM). Morphologies that can be observed under the FESEM include the size of the individual fiber diameter by using the scale bar and also the uniformity in the size of the fibers formed. The surface of the fibers can also be examined under high magnification for the porosity. It can be told from the micrographs that PS nanofibers are not long and continuous (Figure 1(a)), which explains why the nanofibers obtained from PS are fragile and brittle. Beaded structures were also observed (Figure 1(b)). As such, PS nanofibers were not suitable for use in microextraction (i.e., they disintegrated upon agitation under stirring).

**Figure 2:**
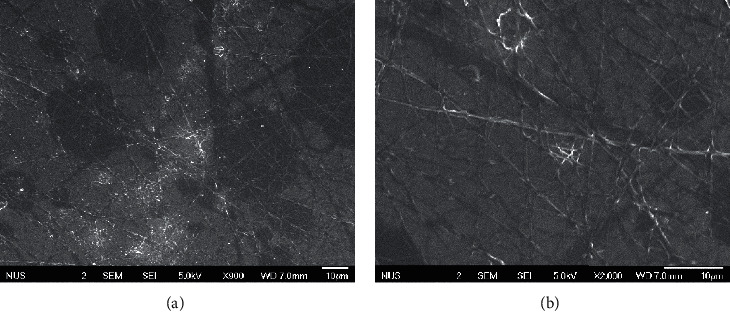
Scanning electron micrographs of PAA nanofibers at (a) ×900 and (b) ×2,000.

For PVP nanofibers, the fibers dissolved too quickly before any SEM images can be taken. Scanning electron micrographs of PAA nanofibers produced from 6 wt. % in DMF polymer solution were taken (Figure 2) before they began to dissolve. The nanofibers formed were very fine and sticky and were unsuitable for use in microextraction.

**Figure 3:**
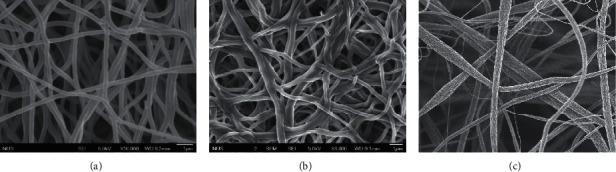
Scanning electron micrograph of (a) PVA nanofibers, (b) PVA/HEC nanofibers, and (c) PVC nanofibers.

The electrospinning conditions stated earlier result from their higher elasticity and ability to form membranes that remain. The duration for electrospinning of all the nanostructured membranes was maintained at 60 min. Even though the polymer solution's flow rate for PVA and PVA/HEC (10 *μ*L/min) was lower than PVC, the thickness of the membranes obtained was relatively constant.  Figure 3 shows the scanning electron micrographs of the nanostructured membranes used in this project under various magnifications.

**Figure 4:**
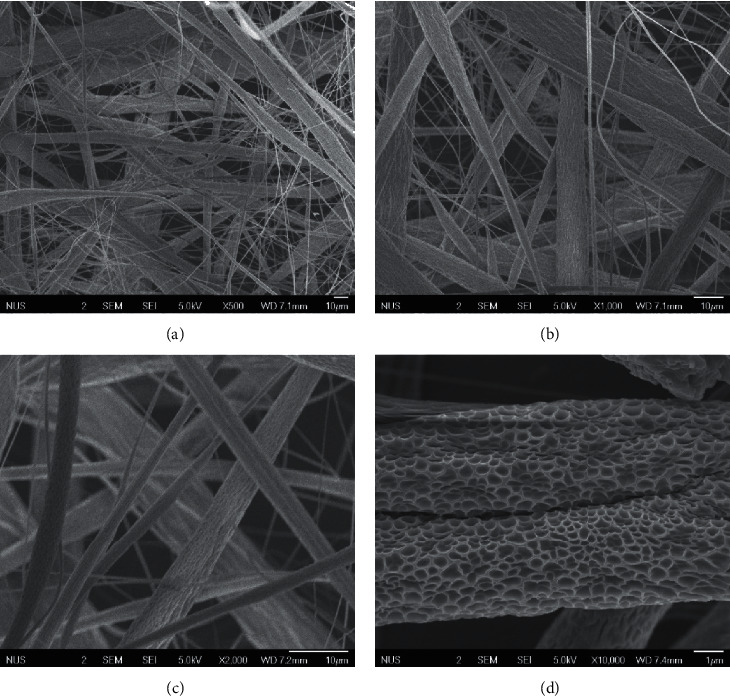
Scanning electron micrographs of PVC nanofibers at magnifications (a) ×500, (b) ×1,000, (c) ×2,000, and (d) ×10,000.

Looking at the scanning electron micrograph of PVC nanofibers (Figure 4), it can be told from the higher-magnification (×10,000) micrograph that PVC nanofibers are highly porous which gives them a higher surface area for interaction with acrylamide.

### 3.2. Optimization of Microextraction Conditions

Solid pet food samples were homogenized with ultrapure water and filtered and the filtered samples were used for optimization. The extraction conditions that affect the extraction efficiency were optimized one after another.

#### 3.2.1. Selection of the Nanostructured Membrane as Sorbent Material

**Figure 5:**
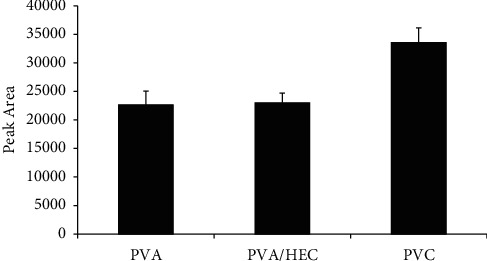
Effect of the nanostructured membrane type on extraction efficiency. Extraction conditions: extraction time 30 min, desorption time 30 min with 0.01 M sulfuric acid as the desorption solvent, and sample volume 10 mL; no adjustment of sample pH.

Extraction was first performed according to the conditions stated in Section 2.5. The first parameter to optimize was the choice of a suitable nanostructured membrane as a sorbent material. Three different nanostructured membranes from different polymer solutions were investigated. To maintain consistency in the extraction process, the weight of the membrane was kept constant. The extraction efficiency of the five different nanostructured membranes is summarized and shown in Figure 5.

The PVC nanostructured membrane showed the highest extraction efficiency compared to the other membranes. The possible extraction mechanism might be the electrostatic interactions between chlorine in PVC and the nitrogen moiety in acrylamide. In the case of PVA and PVA/HEC membranes, hydrogen bonding might play a role in the extraction of acrylamide. Given the higher amount of water molecules present in the sample solution, it is inevitable that water molecules compete with acrylamide molecules for hydrogen-bonding sites with the polymer. Hence, the extraction efficiency of acrylamide was lowered for the PVA and PVA/HEC nanostructured membranes.

#### 3.2.2. Extraction Time

**Figure 6:**
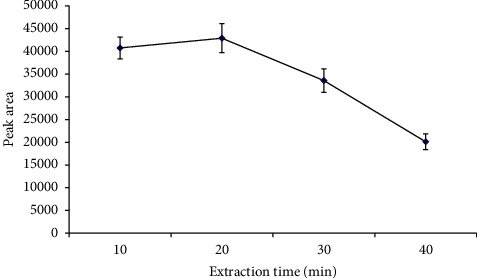
Effect of extraction time on extraction efficiency. Extraction conditions: PVC nanostructured membrane as the sorbent, desorption time 30 min with 0.01 M sulfuric acid as the desorption solvent, and sample volume 10 mL; no adjustment of sample pH.

As microextraction is equilibrium-based, the amount of time needed for equilibrium to establish has to be studied. The extraction time effect on the extraction efficiency was investigated by observing the peak area with an extraction time of 10, 20, 30, and 40 min. The analyte partition coefficient between the aqueous sample and the nanostructured membrane is an essential factor in determining the amount of analyte extracted by the nanostructured membrane. The extraction time profile (Figure 6) shows that the extraction efficiency directly proportional to peak area is optimum at an extraction time of 20 min and decreases subsequently with increased extraction time. One proposed explanation could be that, after the membrane has reached its maximum capacity at a longer extraction time, analytes might be desorbed from the nanostructured membrane, causing a decreased extraction efficiency. Similar behavior has been reported in many microextraction procedures. Another possible explanation can be attributed to the nanostructured membrane physical property: the nanofibers within the membrane are not firmly held together. Hence, the nanostructured membrane cannot withstand a more extended period of agitation, as a result, causing the extraction efficiency to be significantly reduced.

#### 3.2.3. Effect of Sample Volume

**Figure 7:**
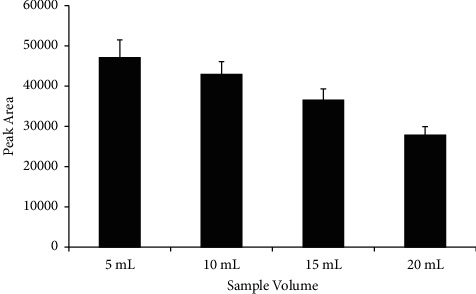
Effect of sample volume on extraction efficiency. Extraction conditions: extraction time 20 min and desorption time 30 min with 0.01 M sulfuric acid as the desorption solvent; no adjustment of sample pH.

Figure 7 shows the effect of sample volume on extraction efficiency. It was observed that, with a larger sample volume, extraction efficiency decreased. As mentioned in the previous section, microextraction is based on equilibrium. In this case, at a larger sample volume, equilibrium may take a longer time to establish due to a higher amount of time needed for mass transfer with a higher amount of analytes. On the contrary, at a smaller sample volume, equilibrium is established much faster and within the selected extraction time of 20 min. Besides, there is also a higher chance for interactions between acrylamide and the PVC molecule to occur at a lower volume (i.e., a more crowded environment). Therefore, 5 mL is the chosen sample volume to be used for this project.

#### 3.2.4. Effect of Desorption Time

**Figure 8:**
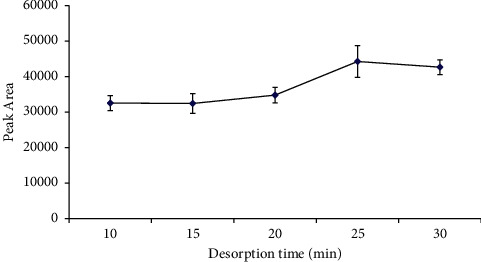
Effect of desorption time on desorption efficiency. Extraction conditions: extraction time 20 min with 0.01 M sulfuric acid as the desorption solvent and sample volume 5 mL; no adjustment of sample pH.

After extraction, it was necessary to desorb the analyte of interest in order to analyze the amount of analyte extracted. As such, desorption time also played a part in obtaining a higher possible concentration of the analyte in the desorption solvent. Desorption time over the range of 10–30 min was investigated, and based on the desorption time profile (Figure 8), peak areas for desorption time from 10 to 20 min increased slowly. Hence, it can be concluded that desorption was incomplete when shorter times were used. At 25 min, acrylamide was almost entirely desorbed. This is because, at 30 min, no considerable increase in peak area was observed. Therefore, 25 min is the optimum time for the analyte desorption after extraction was done.

#### 3.2.5. Effect of Sample pH and Desorption Solvent

**Figure 9:**
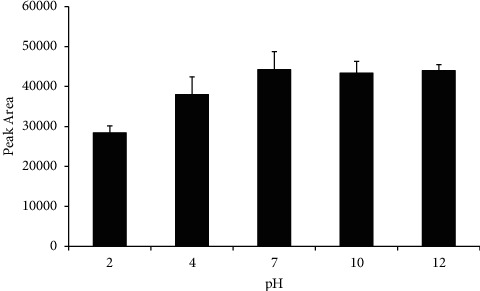
Effect of sample pH on extraction efficiency. Extraction conditions: PVC nanostructured membrane as sorbent material, extraction time 20 min, desorption time 25 min with 0.01 M sulfuric acid as the desorption solvent, and sample volume 5 mL.

To maximize the amount of analyte being extracted, another factor to investigate would be the effect of pH on extraction. Both acidic pH and alkaline pH were experimented with for this part of the experiment. The effect of sample pH on extraction efficiency is shown in Figure 9.

It was observed that, at low pH (i.e., acidic medium), extraction efficiency is lowered, while there is no significant change at high pH (i.e., basic medium). Being an amide, acrylamide protonated at low pH, and thus, it prefers to stay in water as an ionic compound rather than interacting with PVC. There was no effect on extraction efficiency at higher pH, and hence, for the subsequent experiments, no adjustment of pH was needed.

**Figure 10:**
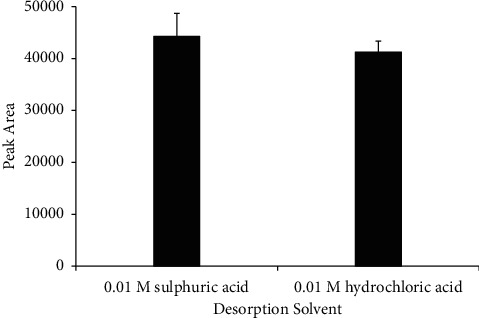
Effect of the desorption solvent on desorption efficiency. Extraction time 20 min, desorption time 25 min, and sample volume 5 mL; no adjustment of sample pH.

The choice of desorption solvent to use was investigated by changing the desorption solvent used. Higher acrylamide was desorbed in 0.01 M sulfuric acid than 0.01 M hydrochloric acid (Figure 10). This could be due to the higher affinity of acrylamide for sulfuric acid than hydrochloric acid.

### 3.3. Method Validation

To assess the method, repeatability, linearity, and limits of detection (LOD) were investigated. As discussed in Section 3.2, the following are the optimized conditions used for the microextraction of acrylamide: PVC nanostructured membrane as sorbent material; extraction time at 20 min; sample volume at 5 mL; desorption time at 25 min; 0.01 M sulfuric acid as the desorption solvent; and no adjustment in sample pH. Before microextraction can be carried out on real samples, a calibration curve from the range of 1 to 100 *μ*g/kg of acrylamide sample solutions was constructed by performing microextraction on the spiked sample with concentrations of 1, 5, 10, 25, 50, 75, and 100 *μ*g/kg.  Table 1 shows the quantitative data obtained through this microextraction method.

The calibration curve had exhibited good linearity with a correlation coefficient (*r*) value of 0.996 for the concentration range 1–100 *μ*g/kg. The final relative standard deviation (RSD) of 5.8% was taken as the RSD average from every concentration with three replicates (*n* = 3). The relatively small value in the RSD indicates that this microextraction method has good reproducibility.

Limits of detection (LOD) are taken as the concentration with a signal-to-noise ratio of 3 (S/N = 3). The LOD in this experiment is 0.008 *μ*g/kg, which is sufficient for the detection of acrylamide in actual samples. Similarly, the limits of quantification (LOQ) are taken as the concentration with a signal-to-noise ratio of 10 (S/N = 10).

The enrichment factor is 180-fold, which was calculated by taking the peak area before and after extraction.

### 3.4. Application to Pet Food and Recovery Test

Four different pet foods were purchased from local supermarkets, processed according to Section 2.4, followed by the extraction procedure according to Section 2.5. Experiments were performed in triplicates (*n* = 3). The amount of acrylamide detected in unspiked solutions of the pet foods is reported in Table 2, before and after considering all the dilution factors during sample preparation.

As there are no reports which state the level of acrylamide intake in animals sufficient to cause toxicity, it is relatively early to conclude whether the amounts of acrylamide present in these pet foods are acceptable or not. However, compared to other food sources such as fried potatoes, bread, and biscuits, the amount of acrylamide detected in these pet foods is considerably lower.

Besides investigating the acrylamide levels present in unspiked sample solutions of the pet foods, it is also necessary to investigate the matrix effect. Two different concentrations (5 and 10 *μ*g/kg) of acrylamide were spiked, and the extraction recoveries and repeatability were evaluated. As usual, the experiment was performed in triplicate (*n* = 2). The results for the relative recoveries and RSD values are listed in Table 2. Relative recoveries for all the samples were favorable, with values greater than 79.7%. The RSD values were between 4.2 and 12.3%.

**Figure 11:**
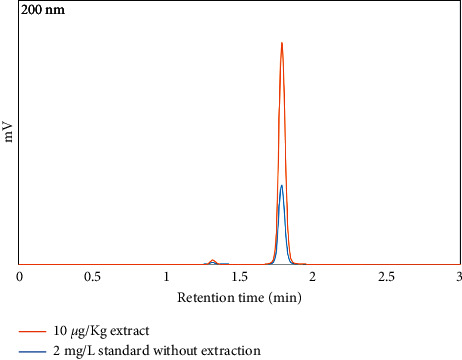
A comparative HPLC chromatogram of standard acrylamide 2 mg/L and the spiked (10 *μ*g/kg) pet food sample extraction.

These results had demonstrated that real food matrices had little effect on the extraction efficiency of membrane microextraction, and the method had good selectivity for the analyte. Hence, it is suitable for the use of acrylamide analysis in actual food samples.  Figure 11 shows the comparison between standard acrylamide 2 mg/L and the spiked 10 *μ*g/kg pet food sample. The chromatogram demonstrates the extractability and efficiency of the PVC electrospun-assisted method on pet food samples.

Acrylamide is formed naturally at high temperatures in food/feedstuff rich in starch. Conventional liquid-liquid extraction followed by sample cleanup is the traditional method adopted in the industry [23]. The LLE method generates a large amount of toxic and expensive solvents. Thus, the cost of analyzing pet food samples is high, and search for cheaper methods for pet food samples is in demand. Recently, a simple electrochemical square-wave voltammetry method has been reported [24]. High detection limits are registered for the electrochemical process. The nanofiber extraction method is simple, cost-effective, and reusable for multiple extractions, and the small sample volume is sufficient to obtain low detection limits.

## 4. Conclusion

Three different nanostructured membranes were successfully prepared by the electrospinning procedure and utilized as a sorbent. A microextraction procedure was developed to quantitate acrylamide in pet food samples. The method showed recoveries (>79.7%) with low detection limits of parts per billion concentration range for complex pet samples. The polyvinyl chloride electrospun nanostructure membrane showed better performance over other membranes. The study strengthens the use of membranes for multiple applications. The PVC electrospun membranes were stable and reusable; no loss and no carryover were observed up to 20 times. This method can be further developed by producing different nanostructured membranes from different polymer solutions to extract other analytes present in food samples or our environment.

## Figures and Tables

**Table 1:** Quantitative data: enrichment factor, linearity, regression equation, correlation coefficient (*r*), repeatability (% RSD, *n* = 3), LOD (S/N = 3), and LOQ (S/N = 10).

Enrichment factor	Linearity range (*μ*g/kg)	Regression equation	Correlation coefficient (r)	% RSD (*n* = 3)	LOD (*μ*g/kg)	LOQ (*μ*g/kg)
180	1–100	*y* = 722.36*x* + 4361.2	0.996	5.8	0.008	0.027

**Table 2:** Concentration of acrylamide from real samples and the extraction recoveries by nanostructured membrane microextraction.

Sample	Concentration in dry food (*μ*g/kg)	Relative recovery (%), spiked at 5 *μ*g/kg	% RSD (*n* = 3)	Relative recovery (%), spiked at 10 *μ*g/kg	% RSD (*n* = 3)
Brand A	0.33	93.7	5.6	79.7	5.9
Brand B	0.52	96.3	9.2	90.9	10.4
Brand C	0.72	113.9	4.2	106.3	6.7
Brand D	0.61	101.8	12.3	106.2	5.2

## Data Availability

The data used to support the findings of this study are included within the article.
